# Fracture de Kocher-Lorenz

**DOI:** 10.11604/pamj.2014.18.299.5104

**Published:** 2014-08-14

**Authors:** Aniss Chagou, Younes Ouchrif

**Affiliations:** 1 Service de Chirurgie Orthopédique et Traumatologique, Centre Hospitalier Universitaire Avicenne, Rabat, Maroc

**Keywords:** Fracture de Kocher-Lorenz, fracture diacondylienne, humérus, Kocher-Lorenz fracture, bicondylar fracture, humerus

## Image en medicine

Les fractures articulaires frontales de l’épiphyse distale de l'humérus sont souvent désignées par le terme vague de fractures du capitellum. Ce sont des fractures rares; elles représententprès de 13% des fractures de cette extrémité osseuse. Parmi ces types fracturaires, la fracture diacondylienne ou fracture de Kocher-Lorenz représente une variété encore plus rare. Nous rapportons le cas d'un patient de 30 ans victime d'une chute directe sur le coude droit en flexion. Le patient s'est présentée aux urgences pour douleur et impotence fonctionnelle. La radiographie du coude (A) a objectivé une fracture diacondylienne avec trait de fracture séparant la trochlée et le condyle sans lésions associées. Une TDM (B) a été demandée pour une meilleure étude de la fracture avant ostéosynthèse. Elle permet également d’éliminer les autres diagnostics différentiels qui sont d'autres lésions à traits de fractures frontales, celle du capitellum, de Hahn Steinthal ou bien les fractures diacolumnaires. Nous avons eu recours à une voie d'abord postérieure avec ostéotomie de l'olécrane avec fixation interne par trois vis antéro postérieure et deux vis obliques. Les pas de vis ont été enfouis de telle sorte qu'ils ne soient plus proéminents dans l'articulation. Une radiographie de contrôle a été réalisée (C). La rééducation a été commencée une semaine après l'intervention. L’évaluation clinique a été réalisée après 5 ans de recul selon l'index de performance du coude décrit par Morrey et al, le résultat a été jugé excellent avec un score de 91 sur 100.

**Figure 1:**
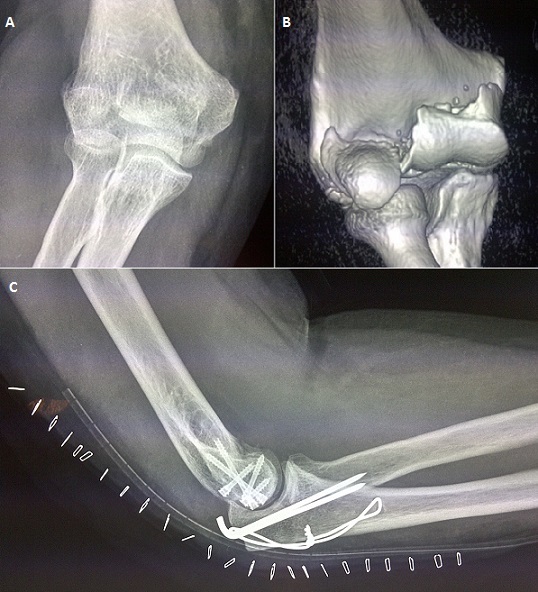
A) radiographie du coudemontrant la fracture frontaleemportanttoute la surface articulaire; B) reconstruction en 3D des images scanographiquesmontrant le trait de fracture frontal. Un deuxième trait scinde la surface articulaire en deux, condyle ettrochlée; C)radiographie de contrôle, abord par ostéotomie de l'olécraneetostéosynthèse par vissage

